# Clinical Efficacy Evaluation of 1-Year Subcutaneous Immunotherapy for *Artemisia sieversiana* Pollen Allergic Rhinitis by Serum Metabolomics

**DOI:** 10.3389/fphar.2020.00305

**Published:** 2020-03-18

**Authors:** Hai-Yun Shi, Chen Pan, Ting-Ting Ma, Yan-Lei Chen, Wei-Jun Yan, Jian-Guo Liu, Meng-Da Cao, Hong-Dong Huang, De-Yun Wang, Xue-Yan Wang, Ji-Fu Wei

**Affiliations:** ^1^Department of Allergy, Beijing Shijitan Hospital, Capital Medical University, Beijing, China; ^2^The First Affiliated Hospital of Nanjing Medical University, Nanjing, China; ^3^School of Basic Medicine and Clinical Pharmacy, China Pharmaceutical University, Nanjing, China; ^4^Duolun People’s Hospital, Inner Mongolia, China; ^5^Department of Nephrology, Beijing Friendship Hospital, Faculty of Kidney Diseases, Capital Medical University, Beijing, China; ^6^Department of Otolaryngology, Yong Loo Lin School of Medicine, National University of Singapore, Singapore, Singapore

**Keywords:** subcutaneous immunotherapy, clinical efficacy, seasonal allergic rhinitis, metabolomics, LC-MS, GC-MS

## Abstract

Subcutaneous immunotherapy is the only treatment that improves the natural progression of allergic rhinitis and maintains long-term outcomes after discontinuation of the drug. Metabolomics is increasingly applied in the study of allergic diseases, including allergic rhinitis. However, little is known about the discovery of metabolites that can evaluate clinical efficacy and possible mechanisms of *Artemisia sieversiana* pollen subcutaneous immunotherapy. Thirty-three patients with *Artemisia sieversiana* pollen allergic rhinitis significantly improved after 1-year subcutaneous immunotherapy treatment, while ten patients were ineffective. Pre- and post-treatment serum samples from these patients were analyzed by metabolomics based on the combined detection of liquid chromatography-mass spectrometry and gas chromatography-mass spectrometry. As a result, L-Tyrosine can be a potential biomarker because of its opposite trend in effective patients and ineffective patients. And mechanism of immunotherapy may be closely related to NO and nitric oxide synthase. The discovery of potential biomarkers and metabolic pathways has contributed to the in-depth study of mechanisms of subcutaneous immunotherapy treatment of *Artemisia sieversiana* pollen allergic rhinitis.

## Introduction

Allergic rhinitis is a type I allergic disease of the nasal mucosa, characterized by paroxysmal repetitive sneezing, watery rhinorrhea, and nasal blockage (Okubo et al., 2017). Allergic rhinitis affects all age groups, increasing in prevalence globally (Wang et al., 2016). Recent studies showed that allergic rhinitis was thought to affect up to 10–40% of the worldwide population (Ma et al., 2014; Song et al., 2015; Bachmann and Kündig, 2017; Wang et al., 2017, 2018). The prevalence of allergic rhinitis in the United States was 19.9% (Wallace and Dykewicz, 2017), while that in adults in Europe ranged from 17 to 28.5% (Brożek et al., 2017; Zheng et al., 2018). The results of a latest meta-analysis indicated that the incidences of allergic rhinitis were 15.79% in Chinese children and 13.26% in Chinese adult (Hu S. J. et al., 2017).

Allergic rhinitis is classified into perennial and seasonal allergic rhinitis. Pollinosis is seasonal allergic rhinitis caused by pollen antigens, frequently complicated by allergic conjunctivitis (Okubo et al., 2017). *Artemisia sieversiana* (Artemisia siversiana Ehrh. ex Willd.) pollen is one of the most common outdoor allergens in China, especially in the north of China. It is also one of the main causes of seasonal allergic rhinitis (Tang et al., 2015; Hu W. H. et al., 2017; Wang et al., 2018). *Artemisia sieversiana* pollen accounted for 36.7%∼50.5% of the pollen content from August to September in Beijing (Wang et al., 2017). A cross-sectional study has shown that about 11.3% of patients in China with respiratory allergies were sensitized to *Artemisia sieversiana* pollen (Gao et al., 2019). This value was much higher in Northern China (78.6%) (Cui and Yin, 2018).

During the summer and autumn pollen season of *Artemisia sieversiana*, seasonal allergic rhinitis of *Artemisia sieversiana* pollen brings inconvenience to patients, such as nasal itching, sneezing, rhinorrhea, nasal congestion, even inducing asthma (Brożek et al., 2017). Currently, the clinical treatment of seasonal allergic rhinitis includes non-specific therapy and SIT. Non-specific therapy mainly refers to drug treatment, such as antihistamines (H2 receptor antagonists), leukotriene receptor blockers, corticosteroids and so on (Greiner et al., 2011). There are two main forms of SIT: SCIT and sublingual allergen-specific immunotherapy (SLIT). However, it is difficult to improve the quality of life of patients and keep therapeutic control (Wallace and Dykewicz, 2017) for lifelong time through drug treatment. Only immunotherapy with individually targeted allergens can alter the natural history of allergic rhinitis (Jacobsen et al., 2007), improve symptom and eliminate the causes of disease, especially suitable for patients with severe or moderate seasonal allergic rhinitis (Cox et al., 2011; Greenhawt et al., 2017).

Subcutaneous immunotherapy was the first applied treatment method of SIT (Cox et al., 2011). During the clinical application for more than one century, SCIT has proved to be well safety under standardized regimens and the professional operation of professionals (Passalacqua et al., 2016). By repeatedly injecting a specific allergen extraction subcutaneously, the patient can reduce or eliminate allergic rhinitis symptoms, and has long-term effects after stopping the drug. SCIT acts by deeply affecting the immunologic allergen-oriented response at various levels (Noon, 1911). SCIT generally takes 3 years and can produce significant and stable symptom improvement after 1-year treatment (Yukselen et al., 2012). The current indicators for the evaluation of SCIT efficacy are subjective indicators. Lack of recognized objective indicators makes the evaluation of SCIT efficacy have certain limitations. Simultaneously, the detailed mechanisms are not clear (Polosa et al., 2004).

Metabolomics is a powerful exploratory tool for discovering interactions between different biochemical molecules and pathways of disease or drugs (Omabe et al., 2018), advancing our understanding of disease progression and drug effects. It was also widely used for the study of allergic diseases. Chen et al. (2019) found that baicalin has protective effects on allergic rhinitis rats by inhibiting the release of immunoglobulin E, histamine, interleukin (IL)-1β, IL-4, and IL-6 by metabolomics studies. Zhuang et al. (2018) found that the potential metabolic pathways of Xanthii Fructus in treating allergic rhinitis in mice include glycerophospholipid metabolism and branched-chain amino acid metabolism. Immunotherapy often affects human metabolism, leading to metabolic disorders (Johnson et al., 2016). However, there are few reports identifying the potential mechanism of SCIT in the treatment of allergic rhinitis by metabolomics. In the present study, we tried to investigate metabolite changes and metabolic activities of *Artemisia sieversiana* pollen allergic rhinitis patients after SCIT and speculate related cellular signaling pathways and epigenetics by metabolomics.

## Materials and Methods

### Patients and Study Design

Seventy-eight patients with *Artemisia sieversiana* pollen allergic rhinitis were recruited in Beijing Shijitan Hospital, Affiliated to Capital Medical University, from July 28 to 31, 2016. Inclusion criteria: course of disease more than 1 year. Typical symptoms appear in the summer and autumn pollen season, and asymptomatic or mild symptoms in the non-pollen season (non-pollen season VAS < 3). Results of intradermal test of *Artemisia sieversiana* pollen allergen ≥+++, and sIgE ≥ II (Uni-CAP allergen-specific IgE detection system). Other types of allergen skin test negative, or “+” and above (including “+”) but specific IgE < II level (cat and dog allergens, even if the skin test and specific IgE do not meet the above conditions, as long as the family does not keep pets Can also be selected). Other sage pollen allergen-specific IgE is 2 or higher than wormwood pollen. Patients received SCIT treatment with standardized *Artemisia sieversiana* pollen allergen extraction purchased from Beijing Macro-Union Pharmaceutical Limited Corporation, Beijing, China (batch number: S20130001, total protein content 1.75 mg/5mL). Standard extraction process for allergens: buffered saline extract and raw materials are mixed to extract allergens, filtered by pH, filtered, prepared, packaged, and tested to obtain the finished allergen extract prickly liquid. The main component of the pricking liquid is a soluble protein mixture in the pollen of *Artemisia sieversiana*. All the patients received standardized regimens and protocols, under operation of professionals in hospital. Patients’ diaries were regularly completed, including TNSS, RQLQ, VAS scores, olfactory function grades and allergic conjunctivitis symptoms scores. The SCIT treatment lasted 1 year.

Thirty-two people fell off during the treatment. The remaining patients (*n* = 46) were evaluated for 1-year efficacy based on comprehensive objective indicator. The curative effect was evaluated by symptoms and sign scores. The curative effect index (%) = (pre-treatment total score − post-treatment total score)/pre-treatment total score × 100% (Gu and Dong, 2005). Patients with effective therapeutic index (≥66%, significantly improved, *n* = 33) were included the effective group. Patients with ineffective therapeutic index (≤25%, *n* = 10) were included the ineffective group and admitted as negative control. The remaining patients (>25%, <66%, not significantly effective or ineffective, *n* = 3) were not included in the analysis because their treatment results were not clear.

### Ethics Statement

The study protocol was in accordance with the ethical standards of the Declarations of Helsinki. The protocol of this study was approved by the Institutional Review Board of Beijing Shijitan Hospital, Affiliated to Capital Medical University, Beijing, China (Z161100000516006, November 2, 2015), and written informed consent was obtained from all subjects.

### Serum Sample Collection

Serum samples pre-treatment and post-treatment were collected from both effective group (*n* = 33) and ineffective group patients (*n* = 10). Approximately 4 mL of peripheral venous blood of the patients was collected with EDTA anticoagulation tubes and immediately centrifuged at 1,500 rpm (10 min, 4°C). Serum was separated by centrifugation, dispensed at 200 μL/tube, and stored at −70°C until serum metabolomics analysis. The samples needed to be processed within 1 h after separation from human body.

### Sample Preparation of Liquid Chromatography-Mass Spectrometry (LC-MS)

Take 20 μL of serum, add 225 μL of ice methanol, vortex for 10 s. Add 750 μL ice methyl tert-butyl ether (MTBE), vortex for 10 s. Shake at 4°C for 10 min. Add 188 μL of ultrapure water, vortex for 20 s. Centrifuge at 14,000 rpm for 2 min at 4°C. 350 μL of the supernatant was pipetted into a 1.5 mL centrifuge tube, spin dried, and placed at −20°C for testing. The remaining liquid was centrifuged for 2 min again, 125 μL of the lower layer was aspirated, evaporated, and placed at −20°C for testing. Add the upper layer of the sample to 110 μL of methanol: toluene (9:1). Vortex for 10 min, sonicate for 10 min, centrifuge at 18,000 rpm for 10 min. The lower layer of the dried sample was vortexed with 60 μL of acetonitrile: water (4:1) for 10 min, sonicated for 10 min, centrifuged at 14,000 rpm for 10 min. The positive ion mobile phase was A (acetonitrile: water = 60:40, 10 mmol ammonium formate, 0.1% formic acid) and B (isopropanol: acetonitrile = 70:30, 10 mmol of ammonium formate, 0.1% formic acid). The anion mobile phase was A (acetonitrile: water = 60:40, 10 mmol ammonium acetate) and B (isopropanol: acetonitrile = 90:10, 10 mmol ammonium acetate).

### Sample Preparation of Gas Chromatography-Mass Spectrometry (GC-MS)

Add 30 μL heptadecanoic acid (0.3 mg/mL) and 400 μL methanol to 50 μL serum, followed by vortexing and centrifugation at 13,000 rpm for 10 min. 300 μL of the supernatant was taken and dried with nitrogen. Add 500 μL of methoxyamine pyridine solution (15 mg/mL), react at 60°C for 2 h. Add 60 μL *N*,*O*-Bis(trimethylsilyl)trifluoroacetamide (BSTFA) with 1% trimethylchlorosilane (TMCS), react at 70°C for 1 h. The mixture was centrifuged at 13,000 rpm for 10 min at 4°C, and then the supernatant was taken for injection measurement.

### Data Pre-processing

LC-MS and GC-MS spectra were manually phased, baseline-corrected and referenced to TSP at 0.0 ppm, using Bruker Topspin 3.0 software (Bruker GmbH, Karlsruhe, Germany) (Li et al., 2017; Hong et al., 2018). Then they were automatically exported to ASCII files using MestReNova (Version 8.0.1, Mestrelab Research SL), which were then imported into “R” software^1^ to do further phase and baseline correction and peak alignment with an in-house developed R-script (Li et al., 2017; Lv et al., 2017; Zhang et al., 2017). The one-dimensional (1D) spectra were automatically binned between 0.2 and 10 ppm using a dynamic adaptive binning approach for subsequent statistical analysis (Wan et al., 2017). Exclude the regions of the residual water and affected signals. The remaining regions of each spectrum were normalized and mean-centered and Pareto-scaled before further multivariate statistical analysis (Chen et al., 2017).

### Multivariate Analysis

In order to filter out irrelevant effects and maximize the discrimination between intergroup differences, unsupervised PCA and supervised OSC-PLS-DA were applied for multivariate statistical analysis (Xing et al., 2018). The OSC was a technique that applied prior to PLS-DA to filter out unrelated variables that were not concerning the class discrimination so as to minimize the influence of unrelated signals (Chen et al., 2017). To assess the validity of the established OSC-PLS-DA model, a repeated two-fold cross-validation method and permutation test (*n* = 2000) were applied. The validity of the models against over fitting was assessed by the parameters R2Y, and the predictive ability was described by Q2Y (Li et al., 2017). Color-coded loadings plot and S-plot were constructed to reveal variables that contributed to the group separation. The observed statistic *p*-values via permutation testing which were less than 0.05 confirmed the significance of the OPLS-DA model at a 95% confidence level.

### Univariate Analysis

Parametric Student’s *t*-test and non-parametric Mann–Whitney test were performed to evaluate the differences of metabolites between groups. The FC values of the identified metabolites between groups as well as their associated *p*-values adjusted by Benjamini and Hochberg method were calculated (Xu et al., 2017).

### Pathway Analysis

MetPA^2^ and KEGG^3^ were used for pathway analysis to identify biologically meaningful metabolic patterns and relevant pathways based on significant differential metabolites. Significant differential metabolites were selected based on OSC-PLS-DA loading/S-plots and FC plots. KEGG enrichment were analyzed in order to detect the functions and pathways of differentially metabolites (Madhu et al., 2018).

## Results

### Metabolites Identification

The results of LC-MS and GC-MS were identified with metabolomics database. LC-MS detected 98 negative icon compounds and 128 positive icon compounds, while GC-MS detected 131 compounds. The results of detection and metabolite identification for both effective group and ineffective group are listed in Supplementary Tables S1–S3. The combined detection range of the two methods, LC-MS and GC-MS, can reflect the level of metabolites in human body comprehensively. Metabolites were identified by querying public metabolomics databases, such as KEGG (see footnote 3) and HMDB^4^.

### Multivariate Analysis

**FIGURE 1:**
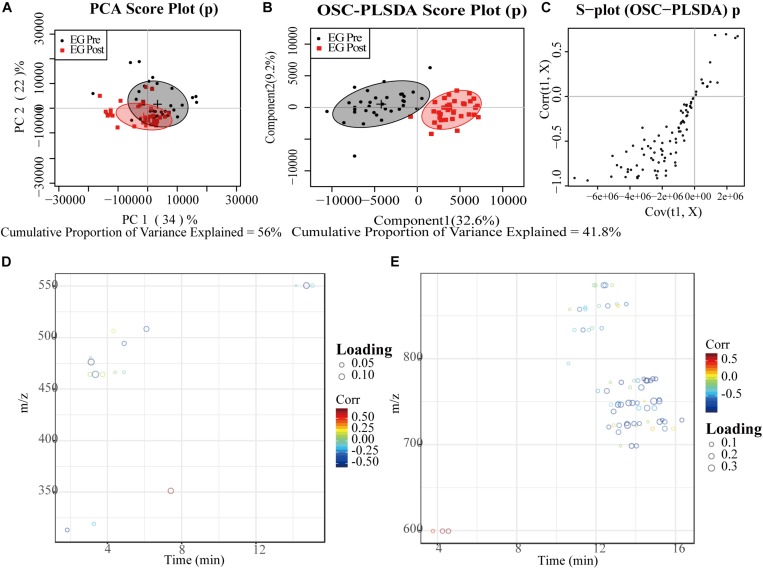
PCA and OPLS-DA analyses of LC-MS for negative ion data from serum of effective patients. **(A)** PCA Score plot; **(B)** OPLS-DA Score plot; **(C)** the corresponding S-plot, points represented differential variables (metabolites): the further away from the center of a variable, the more contribution of the variable to the grouping; **(D,E)** the color-coded loading plots according to the correlation coefficients, from blue to red, the relativity gradually enhanced. EG, effective group.

**FIGURE 2:**
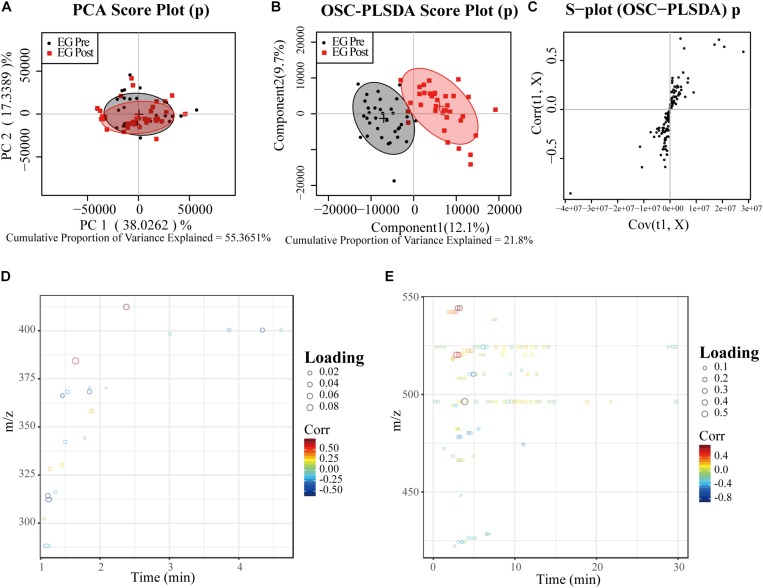
PCA and OPLS-DA analyses of LC-MS for positive ion data from serum of effective patients. **(A)** PCA Score plot; **(B)** OPLS-DA Score plot; **(C)** the corresponding S-plot, points represented differential variables (metabolites): the further away from the center of a variable, the more contribution of the variable to the grouping; **(D,E)** the color-coded loading plots according to the correlation coefficients, from blue to red, the relativity gradually enhanced. EG, effective group.

**FIGURE 3:**
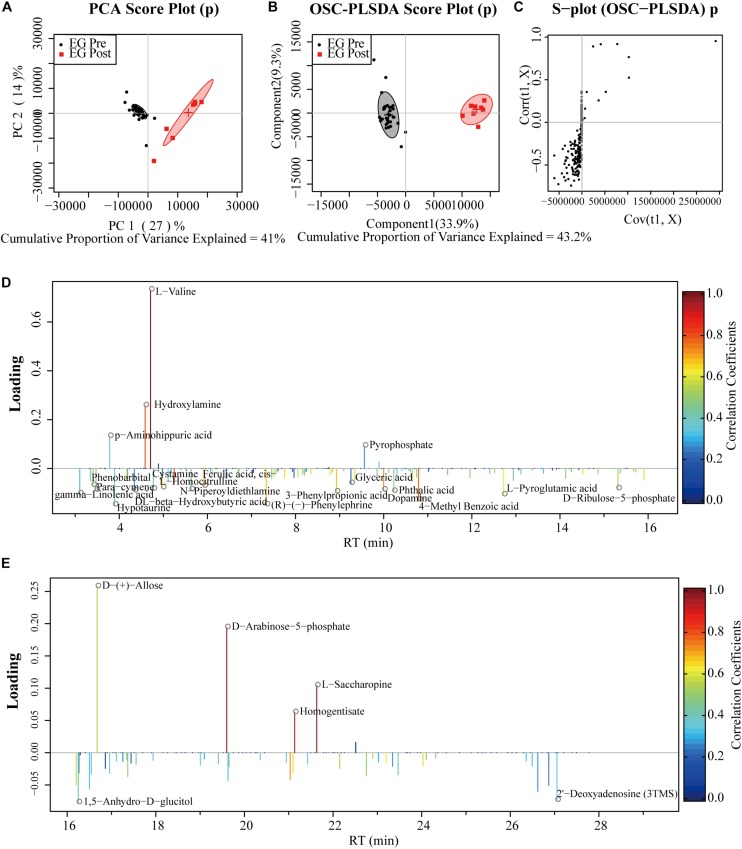
PCA and OPLS-DA analyses of GC-MS data from serum of effective patients. **(A)** PCA Score plot; **(B)** OPLS-DA Score plot; **(C)** the corresponding S-plot, points represented differential variables (metabolites): the further away from the center of a variable, the more contribution of the variable to the grouping; **(D,E)** the color-coded loading plots according to the correlation coefficients, from blue to red, the relativity gradually enhanced. EG, effective group.

Results of LC-MS and GC-MS from effective patients (*n* = 33) were taken for a multivariate analysis. For both LC-MS negative ions and positive ions, the PCA score plots exhibited a severe overlap between pre-treatment (Pre) and post-treatment (Post) (Figures 1A, 2A). Applying supervised OSC-PLS-DA eliminated the variations that were unrelated with the grouping discrimination and achieved better separation between Pre and Post groups (Figures 1B, 2B). In the PCA (Figure 3A) and OPLS-DA score plots (Figure 3B) of GC-MS, the Pre and Post groups were satisfactory separated. In the S-plots (Figures 1C, 2C, 3C), points in different shapes represented differential variables (metabolites): the further away from the center of a variable, the more contribution of the variable to the grouping. The loading plots (Figures 1D,E, 2D,E, 3D,E) were color coded according to the correlation coefficients, from blue to red, the relativity gradually enhanced. They revealed the variation of metabolites in Post group compared with Pre group in serum.

Results of LC-MS and GC-MS from ineffective patients (*n* = 10) were taken for a multivariate analysis using the same method. Supervised OSC-PLS-DA were applied and achieved better separation between Pre and Post groups (Supplementary Figures S1B, S2B, S3B), compared with PCA score plots (Supplementary Figures S1A, S2A, S3A). The color-coded S-plots (Supplementary Figures S1C, S2C, S3C) and loading plots (Supplementary Figures S1D,E, S2D,E, S3D,E) for OPLS-DA revealed the variation of metabolites in Post group, compared with Pre group in serum.

### Univariate Analysis and Comparison Between Effective Group and Ineffective Group

Univariate analysis was also carried out to calculate the relative content of each metabolite in Pre group and Post group among effective patients (*n* = 33) and ineffective patients (*n* = 10) by using R language, and the absolute value of the change rate of each metabolite was obtained. FC was calculated to indicate the degree of variation of differential metabolites.

Among effective patients (*n* = 33), as can be seen in Supplementary Tables S1, S2, LC-MS detected 43 negative ion differential metabolites (*P* < 0.05) and 12 positive ion differential metabolites (*P* < 0.05). In Supplementary Table S3, 108 differential metabolites were detected by GC-MS (*P* < 0.05). Among ineffective patients (*n* = 10), 143 different compounds (*P* < 0.05) were detected by LC-MS and GC-MS. The FC values (Pre vs. Post) of the detected metabolites as well as their associated *P*-values were summarized in Supplementary Tables S1–S3. Red indicates more pre- than post-treatment, meaning the patient’s level of metabolite decreased at the end of SCIT treatment. Blue indicates less pre- than post-treatment, meaning the patient’s metabolite level rose at the end of this treatment. The darker the red or blue color, the greater the drop or increase.

Through the above results, patients in the effective group and the ineffective group had different metabolic level changes after 1-year SCIT treatment. The results of the ineffective group were taken as negative control, compounds with significant changes in the effective group (*P* < 0.05) but no changes (*P* ≥ 0.05) in the ineffective group or with opposite trends were listed separately in Table 1. There existed 54 compounds changing significantly pre-and-post among effective patients while unchanging among ineffective patients. In particular, metabolism level of L-tyrosine among effective patients was increased after 1-year SCIT treatment, while it was decreased in ineffective patients after treatment.

**TABLE 1:**
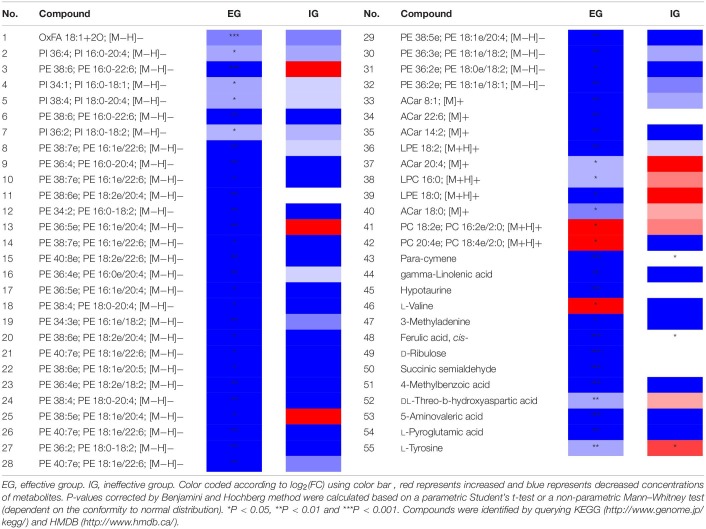
Comparison of potential marker metabolites between effective group and ineffective group.

### Metabolite Pathway Analysis of Effective Group

Based on the above results, significant metabolites in effective group were subjected to pathway analysis using MetPA to explore the most impacted pathways in patients who received 1-year SCIT and achieved effective result.

**FIGURE 4:**
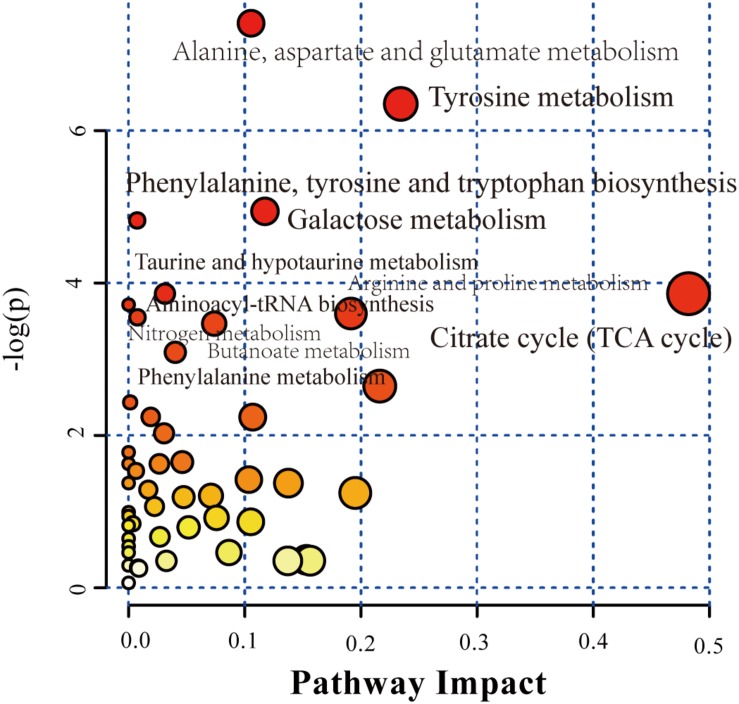
Pathway analysis of significant metabolites in serum of effective patients between Pre and Post groups. Bubble plot of the altered metabolic pathways in the serum of pre group compared with post group. Bubble area is proportional to the impact of each pathway, with color denoting the significance from highest in red to lowest in white. The labels in the figures correspond to KEGG ID.

**FIGURE 5:**
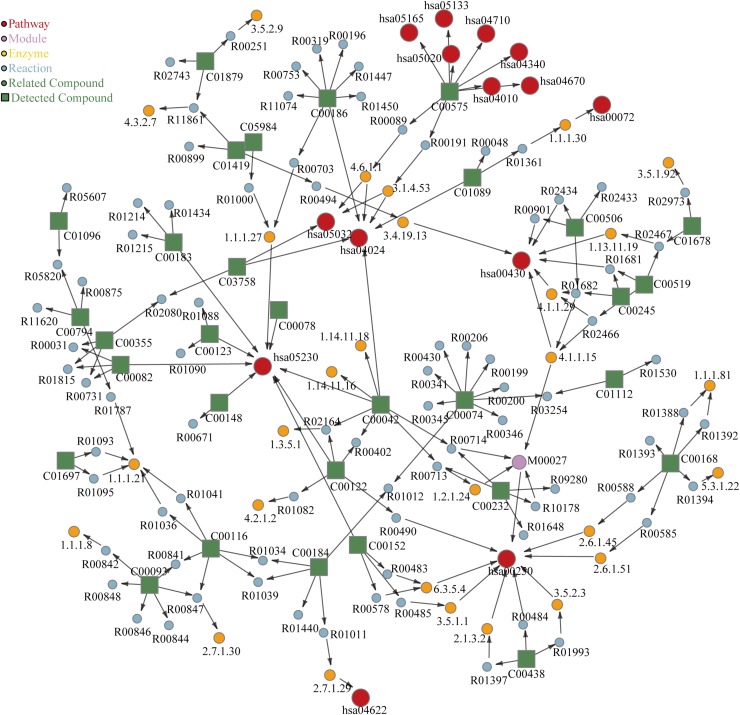
KEGG enrichment of metabolites in serum of effective patients between Pre and Post groups. The labels in the figure corresponds to KEGG ID.

Bubble plot (Figure 4) shown the related metabolic pathways in effective group. There are 11 main metabolic pathways (*P* < 0.05) involved, shown in Table 2 with their related compounds. For pathway enrichment analysis we used the databases provided by KEGG, identified 30 compounds that were significantly enriched (*P* < 0.05), spanning 22 pathways (Figure 5). Red circles refer to KEGG pathways, related modules, enzymes, reactions and compounds were also analyzed.

**TABLE 2:** Pathway analysis and related significant metabolites of effective group.

No.	Pathways	Compounds	KEGG ID	P
1	Alanine, aspartate and glutamate metabolism	L-Asparagine	C00152	***
		Fumaric acid	C00122	
		*N*-Carbamoyl-L-aspartate	C00438	
		Succinic semialdehyde	C00232	
		Succinic acid	C00042	
2	Tyrosine metabolism	L-Tyrosine	C00082	**
		4-Hydroxyphenethyl alcohol	C06044	
		3,4-Dihydroxy-L-phenylalanine	C00355	
		Homogentisate	C00544	
		Dopamine	C03758	
		Fumaric acid	C00122	
3	Galactose metabolism	Glycerol	C00116	**
		D-(-)-Sorbitol	C00794	
		Alpha-Lactose	C00243	
		Galactitol	C01697	
		Myo-Inositol	C00137	
4	Phenylalanine, tyrosine and tryptophan biosynthesis	Phosphoenolpyruvic acid	C00074	**
		3,4-Dihydroxybenzoic acid	C00230	
		L-Tyrosine	C00082	
		L-Tryptophane	C00078	
5	Citrate cycle (TCA cycle)	Succinic acid	C00042	*
		Fumaric acid	C00122	
		Phosphoenolpyruvic acid	C00074	
6	Taurine and hypotaurine metabolism	L-Cysteic acid	C00506	*
		Hypotaurine	C00519	
		Taurine	C00245	
7	Aminoacyl-tRNA biosynthesis	L-Asparagine	C00152	*
		L-Valine	C00183	
		L-Leucine	C00123	
		L-Tryptophane	C00078	
		L-Tyrosine	C00082	
		L-Proline	C00148	
8	Arginine and proline metabolism	Fumaric acid	C00122	*
		L-Proline	C00148	
		Urea	C00086	
		Putrescine	C00134	
		Creatinine	C00791	
		5-Aminovaleric acid	C00431	
9	Nitrogen metabolism	L-Tyrosine	C00082	*
		L-Tryptophane	C00078	
		Taurine	C00245	
		L-Asparagine	C00152	
10	Butanoate metabolism	Succinic semialdehyde	C00232	*
		Succinic acid	C00042	
		Fumaric acid	C00122	
		DL-beta-Hydroxybutyric acid	C01089	
11	Phenylalanine metabolism	L-Tyrosine	C00082	*
		Fumaric acid	C00122	
		Succinic acid	C00042	
		3-Phenylpropionic acid	C05629	

P-values corrected by Benjamini and Hochberg method were calculated based on a parametric Student’s t-test or a non-parametric Mann–Whitney test (dependent on the conformity to normal distribution). *P < 0.05, **P < 0.01 and ***P < 0.001.

## Discussion

Allergic rhinitis caused by pollen is usually seasonal allergic rhinitis with obvious seasonal characteristics. Allergic symptoms occur in the pollen season, but no symptoms appear in the non-pollen seasons, which is thought to be related to pollen exposure and pollen concentration in the air (Wakamiya et al., 2019; Xie et al., 2019). SIT is considered as the only way to eradicate for allergic rhinitis. However, its clinical application still has some shortcomings: there are no convincing objective indicators to evaluate the clinical efficacy; the mechanism of action is too complex and unclear. Metabolites reflect the changes of cell function after disease or external stimulation, especially the related enzymatic or chemical reactions (Schrimpe-Rutledge et al., 2016; Zampieri et al., 2017). Therefore, metabolomics is widely used in complex diseases, such as cancer, cardiovascular disease and diabetes mellitus. It is often used to diagnose diseases, understand disease mechanisms, identify new drug targets, customize drug treatment and monitor therapeutic effects (Wishart, 2016; Newgard, 2017). Allergic diseases are also complex and heterogeneous diseases suitable for metabolomics research (Bunyavanich and Schadt, 2015). Metabolomics has been extensively studied on the effects of metabolism on the progression of allergic diseases, especially asthma (Kelly et al., 2017; Reinke et al., 2017). It has the potential of diagnosis and treatment, and is considered to be an effective method to find biomarkers of allergic diseases (Villaseñor et al., 2017). Up to now, the research on the therapeutic methods of allergic rhinitis based on metabonomics is limited. There were two animal level studies that have found the metabolic mechanism of drug treatment for allergic rhinitis (Zhuang et al., 2018; Chen et al., 2019). However, there is a blank in the metabolomics study of SCIT.

In our study, we recruited 78 allergic rhinitis patients with *Artemisia sieversiana* pollen allergy during the pollen season, and finally 43 were included in the analysis. They were all treated with SCIT for 1 year. In addition to the detailed inclusion criteria in the method above, other covariates, such as genders and fasting or not, were not taken into account, since the current study on human does not indicate the impact of other covariates on the results (Adamko et al., 2018; Ma et al., 2019). Through metabolomics detection and analysis of serum from these patients before and after treatment, we studied the difference of metabolic levels in *Artemisia sieversiana* pollen allergic rhinitis patients before and after 1-year of SCIT treatment by metabonomic method, and combined with the physiological and biochemical significance of different metabolites and related metabolic pathways, we made some positive discoveries.

Firstly, SCIT currently has no objective clinical evaluation indicators or biomarkers. Only subjective indicators such as therapeutic index can be used for the evaluation of curative effect, which makes the clinical evaluation of this therapy lack scientific to a certain extent. Therefore, in our study, by using ineffective patients as negative control, we found metabolites that changed significantly in the effective patients but remained unchanged or opposite in the ineffective patients. Among them, L-tyrosine was increased in the effective patients and decreased in the ineffective patients, which is an opposite trend (Table 1). At the same time, this compound was involved in five metabolic pathways related in effective patients (Table 2). L-Tyrosine is one of the ingredients of MPL adjuvant of allergy vaccine, which assists in redressing a healthy balance between TH1- and TH2-type activities and enhances production of allergen-specific IgG (Patel et al., 2014). Combined with our findings, we can infer that L-tyrosine metabolism in patients may affect the treatment effect, and be a key factor that leads to effective or ineffective treatment result. These phenomena showed that L-tyrosine could be used as a biomarker to evaluate clinical efficacy of SCIT.

On the other hand, through metabolomics analysis of patients who were effective in 1-year SCIT treatment, we found that the levels of various metabolites in the body changed significantly after patients received SCIT (Supplementary Tables S1–S3), involving 11 metabolic pathways (Table 2). These changes are likely to reveal the impact of SCIT treatment on patients and may be related to the mechanism of SCIT.

This study found that there were significant disorders in nitric oxide (NO) related metabolism before and after treatment, including arginine and proline metabolism, tyrosine metabolism, and nitrogen metabolism. NO acts as an inflammatory mediator in the airway (Kim et al., 2016). NO imbalance is considered to play an important role in the pathogenesis of allergic rhinitis, and nasal NO has been proved to be the best biomarker for distinguishing AR from non-AR (Takeno et al., 2014). NO is synthesized from L-arginine by nitric oxide synthase, which also plays an important role in the pathophysiology of rhinitis, especially in the glandular function of allergic nasal mucosa (Kang et al., 2000). Nitric oxide synthase is expressed in many cell types, such as epithelial cells, inflammatory cells (macrophages, neutrophils, and mast cells), airway nerves and vascular endothelial cells (Kim et al., 2016). The recruitment of eosinophils is accompanied by an increase in NO in the nasal cavity, which leads to oxidative stress (Hanazawa et al., 2000). Eosinophils are considered to be responsible for allergic-related inflammatory diseases. Peripheral blood eosinophils are biomarkers of allergic airway inflammation (Amin et al., 2016). Studies have found that fractional exhaled NO and blood eosinophil levels are significantly correlated with index%Tfh2 cells per% Breg cells (Kamekura et al., 2015). High-dose allergen administered resulting in immune deviation from a Th2 to a Th1-driven response is considered to be one of the mechanisms of immunological and clinical tolerance in SIT (Jasper et al., 2017). In perennial allergic rhinitis, inducible nitric oxide synthase in nasal mucosa is up-regulated, and NO in nasal cavity decreases after treatment with topical corticosteroids (Hanazawa et al., 2000). These evidences suggest that SCIT treatment of rhinitis may play a role in the production and activity of NO, thereby improving the symptoms and diseases of patients with rhinitis.

In addition, arginine is a substrate for nitric oxide synthase and arginase. Arginase expression is strongly induced by cytokines, in particular IL-4 and IL-13, which are produced at elevated level in airways and which activate inflammatory pathways. Arginase provides a precursor for polyamines and proline by modulating nitric oxide synthase activity, which stimulate cell growth and collagen synthesis (Lewandowicz and Pawliczak, 2007). Arginase competes with nitric oxide synthase for L-arginine, which catalyzes the hydrolysis of arginine to urea and ornithine (Yasar et al., 2011). Therefore, an increase in serum arginase activity may limit the formation of NO catalyzed by inducible nitric oxide synthase, resulting in allergic rhinitis (Maarsingh et al., 2006). The metabolic disorders of arginine and proline metabolism in this study may be closely related to the effective treatment of SCIT.

In our study, taurine and taurine metabolic levels increased significantly in patients with allergic rhinitis after 1 year of treatment. Taurine and taurine metabolism were also found to increase after asthma treatment (Comhair et al., 2015; Quan-Jun et al., 2017), which may be related to taurine inhibiting the production of inflammatory cytokines, reducing nasal friction and histamine (Nam et al., 2017). TCA cycle (Quan-Jun et al., 2017; Khoo et al., 2018; Chen et al., 2019) and phenylalanine, tyrosine, and tryptophan biosynthesis (Zinkeviciene et al., 2016) has also been found to be of some significance in the study of metabolomics of many allergies. These findings have certain indications and auxiliary effects for researchers to further discover the mechanism of SCIT for allergic rhinitis.

## Conclusion

Our metabolomics studies have found some differential metabolites associated with the SCIT treatment of *Artemisia sieversiana* pollen allergic rhinitis. We found that L-tyrosine could be used as a biomarker to evaluate clinical efficacy of SCIT, because of its opposite trend in effective patients and ineffective patients. At the same time, the metabolic pathways related to NO production and metabolism *in vivo* were obviously disordered after 1 year of treatment for allergic rhinitis. We can infer that the mechanism of immunotherapy may be closely related to NO and nitric oxide synthase. However, further replication studies are necessary to validate our inference. These studies may provide a new perspective with which to find potential biomarkers of and understand the mechanism of SCIT, as well as a new reference for personalized treatment.

## Data Availability Statement

The data used to support the findings of this study are available from the corresponding author upon reasonable request.

## Ethics Statement

The studies involving human participants were reviewed and approved by the Institutional Review Board of Beijing Shijitan Hospital, Affiliated to Capital Medical University. Written informed consent to participate in this study was provided by the participants’ legal guardian/next of kin.

## Author Contributions

H-YS, T-TM, Y-LC, W-JY, and J-GL completed the work of sample collection and patient information collection. CP completed the experiment and data analysis and wrote this thesis. M-DC helped to data analysis. J-FW, X-YW, D-YW, and H-DH conceived of the study and participated in its design and coordination and helped to draft the manuscript. All authors read and approved the final manuscript.

## Conflict of Interest

The authors declare that the research was conducted in the absence of any commercial or financial relationships that could be construed as a potential conflict of interest.

## Footnotes

1 http://cran.r-project.org/

2 http://www.metaboanalyst.ca

3 http://www.genome.jp/kegg/

4 http://www.hmdb.ca/

## Abbreviations

AIT: allergen-specific immunotherapy
FC: fold change
KEGG: Kyoto Encyclopedia of Genes and Genomes
OSC: orthogonal signal correction
OSC-PLS-DA: orthogonal signal correction partial least-squares discriminant analysis
PCA: principal component analysis
PLS-DA: partial least-squares discriminant analysis
RQLQ: rhinoconjunctivitis quality of life questionnaires
SCIT: subcutaneous immunotherapy
SIT: specific immunotherapy
TNSS: total nasal symptom scores.
