# Supplementary material for: Clinical Efficacy Evaluation of 1-Year Subcutaneous Immunotherapy for Artemisia sieversiana Pollen Allergic Rhinitis by Serum Metabolomics
Source: Front Pharmacol. 2020 Mar 18;11:305. doi: 10.3389/fphar.2020.00305 (PMC7093654; doi:10.3389/fphar.2020.00305)
Supplement: Supplementary file 1 [file Data_Sheet_1.docx]

**Supplementary materials**

**Supplementary Figure Legends**

**Figure S1. PCA and OPLS-DA analyses of LC-MS for negative ion data from serum of ineffective patients.**

(A) PCA Score plot; (B) OPLS-DA Score plot; (C) the corresponding S-plot, points represented differential variables (metabolites): the further away from the center of a variable, the more contribution of the variable to the grouping; (D, E) the color-coded loading plots according to the correlation coefficients, from blue to red, the relativity gradually enhanced. IG: Ineffective Group.

**Figure S2. PCA and OPLS-DA analyses of LC-MS for positive ion data from serum of ineffective patients.**

(A) PCA Score plot; (B) OPLS-DA Score plot; (C) the corresponding S-plot, points represented differential variables (metabolites): the further away from the center of a variable, the more contribution of the variable to the grouping; (D, E) the color-coded loading plots according to the correlation coefficients, from blue to red, the relativity gradually enhanced. IG: Ineffective Group.

**Figure S3. PCA and OPLS-DA analyses of GC-MS data from serum of ineffective patients.**

(A) PCA Score plot; (B) OPLS-DA Score plot; (C) the corresponding S-plot, points represented differential variables (metabolites): the further away from the center of a variable, the more contribution of the variable to the grouping; (D, E) the color-coded loading plots according to the correlation coefficients, from blue to red, the relativity gradually enhanced. IG: Ineffective Group.

**Supplementary Figures**

**Figure S1**

**
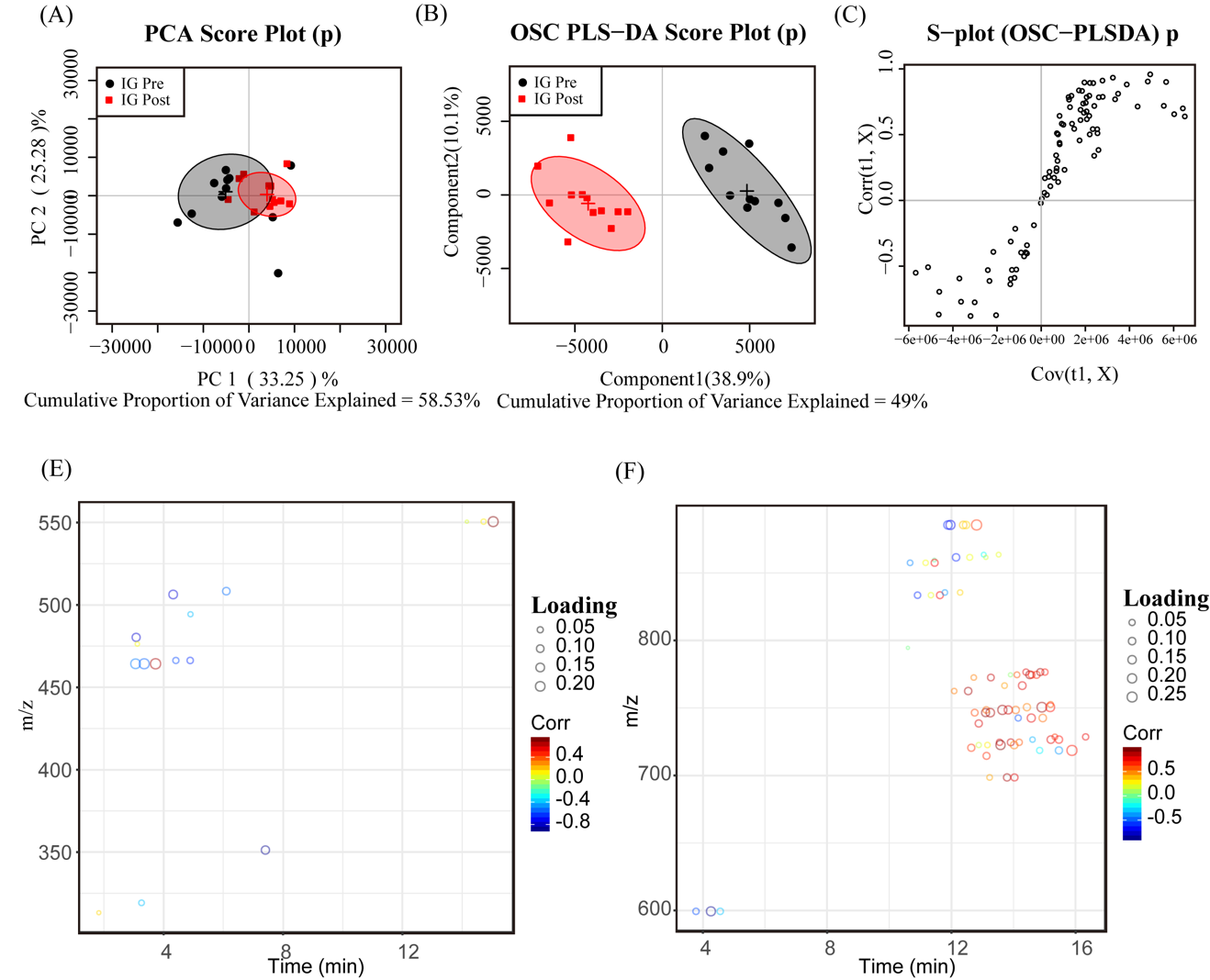
**

**Figure S2**

**
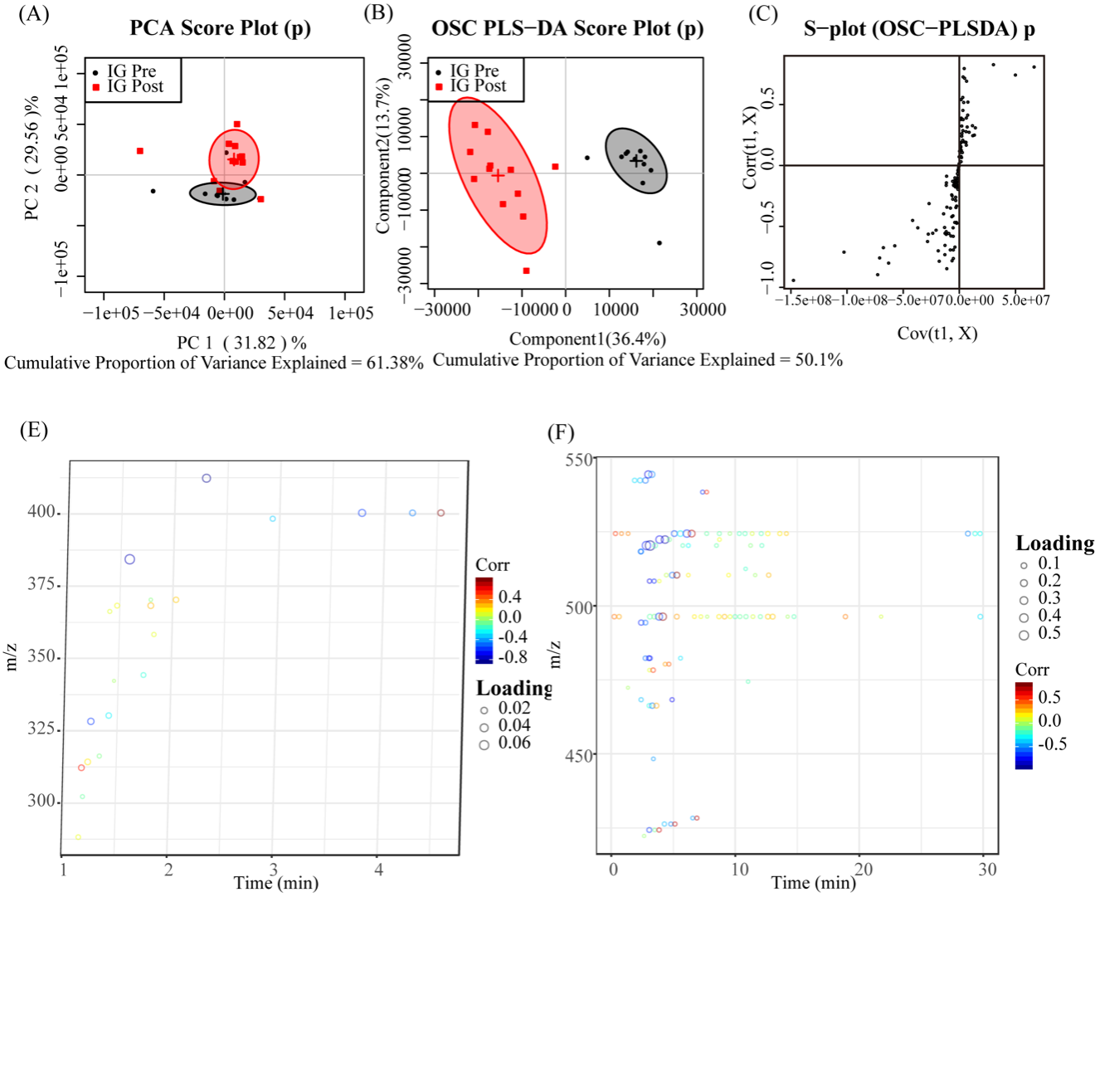
**

**Figure S3**


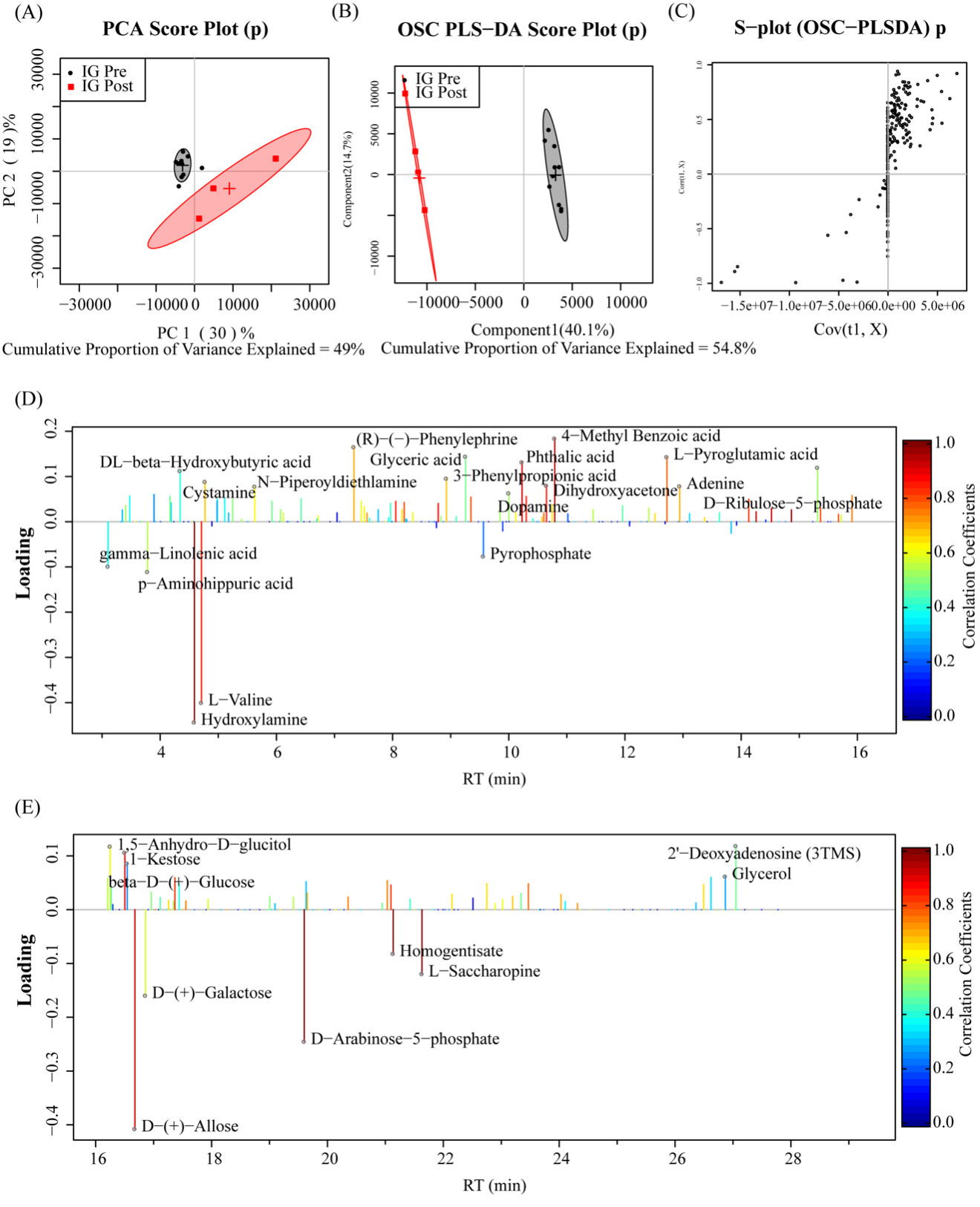


**Table 1 Metabolites and their fold changes from LC-MS negative ion and the associated P-values**

| No. | tR/min | Metabolite name | Effective group (n=33) | | Ineffective group (n=10) | |
| --- | --- | --- | --- | --- | --- | --- |
|  |  |  | FC | P | FC | P |
| 1 | 1.83 | OxFA 18:1+2O; [M-H]- | -0.88 | *** | -0.86 |  |
| 2 | 3.06 | LPE 17:1; [M-H]- | 0.03 |  | 0.54 |  |
| 3 | 3.08 | PE 18:0e; PE 14:0e/4:0; [M-H]- | -2.07E+01 |  | 30.18 |  |
| 4 | 3.12 | LPE 18:2; [M-H]- | -1.38 |  | 0.1 |  |
| 5 | 3.26 | OxFA 20:3+1O(1Cyc); [M-H]- | -0.47 |  | 0.34 |  |
| 6 | 3.35 | LPE 17:1; [M-H]- | -0.16 |  | 0.57 |  |
| 7 | 3.73 | LPE 17:1; [M-H]- | 0.25 |  | -6.47 | * |
| 8 | 3.78 | LPI 18:0; [M-H]- | 26.5 | *** | 27.08 | ** |
| 9 | 4.26 | LPI 18:0; [M-H]- | 2.64 |  | 3.52 |  |
| 10 | 4.32 | PE 20:1e; PE 16:1e/4:0; [M-H]- | 0.23 |  | 1.47 |  |
| 11 | 4.41 | LPE 17:0; [M-H]- | 0.5 |  | 1.77 |  |
| 12 | 4.56 | LPI 18:0; [M-H]- | 3.13 |  | -5.28 |  |
| 13 | 4.89 | LPE 17:0; [M-H]- | 0.13 |  | 1.07 |  |
| 14 | 4.9 | PE 19:0e; PE 16:0e/3:0; [M-H]- | -0.06 |  | 0.62 |  |
| 15 | 6.1 | PE 20:0e; PE 18:0e/2:0; [M-H]- | -0.11 |  | 0.63 |  |
| 16 | 7.41 | FAHFA 21:2; FAHFA 18:2/3:0; [M-H]- | 28.96 | *** | 29.61 | *** |
| 17 | 8.13 | OxFA 18:0+1O; [M-H]- | 1.04 | * | 1.16 | * |
| 18 | 10.6 | PE 42:11e; PE 22:6e/20:5; [M-H]- | -0.31 |  | 0.15 |  |
| 19 | 10.67 | PI 36:4; PI 16:0-20:4; [M-H]- | -0.81 |  | 0.96 |  |
| 20 | 10.91 | PI 34:2; PI 16:0-18:2; [M-H]- | 0.3 |  | 28.8 | * |
| 21 | 11.17 | PI 36:4; PI 16:0-20:4; [M-H]- | -0.68 | * | -0.67 |  |
| 22 | 11.34 | PI 34:2; PI 16:0-18:2; [M-H]- | -0.59 |  | -0.28 |  |
| 23 | 11.45 | PI 36:3; PI 18:1-18:2; [M-H]- | -0.3 |  | -0.35 |  |
| 24 | 11.46 | PI 36:4; PI 16:0-20:4; [M-H]- | -0.63 |  | -7.96 |  |
| 25 | 11.63 | PI 34:2; PI 16:0-18:2; [M-H]- | -0.68 |  | -3.54 |  |
| 26 | 11.79 | PI 34:1; PI 16:0-18:1; [M-H]- | -0.36 |  | 1.19 |  |
| 27 | 11.89 | PI 38:4; PI 18:0-20:4; [M-H]- | -0.6 |  | 5.22 |  |
| 28 | 11.97 | PI 38:4; PI 18:0-20:4; [M-H]- | 1.26 |  | 5.4 |  |
| 29 | 12.09 | PE 38:6; PE 16:0-22:6; [M-H]- | -3.09 | *** | 1.25 |  |
| 30 | 12.15 | PI 36:2; PI 18:0-18:2; [M-H]- | 1.84 |  | 7.18 |  |
| 31 | 12.28 | PI 34:1; PI 16:0-18:1; [M-H]- | -0.38 | * | -0.26 |  |
| 32 | 12.38 | PI 38:4; PI 18:0-20:4; [M-H]- | -0.45 | * | -0.25 |  |
| 33 | 12.43 | PI 38:3; PI 18:0-20:3; [M-H]- | -0.41 |  | -0.41 |  |
| 34 | 12.47 | PI 38:4; PI 18:0-20:4; [M-H]- | -0.45 |  | -0.26 |  |
| 35 | 12.54 | PE 38:6; PE 16:0-22:6; [M-H]- | -2.32 | ** | -1.52 |  |
| 36 | 12.59 | PI 36:2; PI 18:0-18:2; [M-H]- | -0.56 | * | -0.32 |  |
| 37 | 12.64 | PE 36:6e; PE 16:1e/20:5; [M-H]- | -3.9 | *** | -2.74 | * |
| 38 | 12.72 | PE 40:8e; PE 18:2e/22:6; [M-H]- | 0.07 |  | 0.24 |  |
| 39 | 12.75 | PE 38:7e; PE 16:1e/22:6; [M-H]- | -2.3 | ** | -0.12 |  |
| 40 | 12.81 | PI 38:4; PI 18:0-20:4; [M-H]- | 1.21 |  | -5.48 | * |
| 41 | 12.88 | PE 36:5e; PE 16:1e/20:4; [M-H]- | -2.45E+01 | *** | -2.14 | * |
| 42 | 12.88.1 | PE 36:4; PE 16:0-20:4; [M-H]- | -2.35 | ** | -1.16 |  |
| 43 | 12.9 | PI 38:3; PI 18:0-20:3; [M-H]- | -0.04 |  | -0.56 | * |
| 44 | 13.04 | PI 36:1; PI 18:0-18:1; [M-H]- | -0.45 |  | 0.6 |  |
| 45 | 13.1 | PE 38:7e; PE 16:1e/22:6; [M-H]- | -2.23 | * | -1.35 |  |
| 46 | 13.11 | PI 36:2; PI 18:0-18:2; [M-H]- | -0.02 |  | -0.15 |  |
| 47 | 13.12 | PE 38:6e; PE 18:2e/20:4; [M-H]- | -2.12 | ** | 0.07 |  |
| 48 | 13.13 | PE 34:2; PE 16:0-18:2; [M-H]- | -2.04 | ** | -1.33 |  |
| 49 | 13.16 | PE 36:5e; PE 16:1e/20:4; [M-H]- | -2.58 | ** | 5.36 |  |
| 50 | 13.23 | PE 34:3e; PE 16:1e/18:2; [M-H]- | 0 |  | -0.87 |  |
| 51 | 13.25 | PE 38:7e; PE 16:1e/22:6; [M-H]- | -2.22 | * | -1.88 |  |
| 52 | 13.27 | PE 40:8e; PE 18:2e/22:6; [M-H]- | -2.43 | ** | -1.49 |  |
| 53 | 13.52 | PI 36:1; PI 18:0-18:1; [M-H]- | -0.47 |  | 0.12 |  |
| 54 | 13.55 | PE 36:4e; PE 16:0e/20:4; [M-H]- | -2.3 | ** | -0.32 |  |
| 55 | 13.58 | PE 36:5e; PE 16:1e/20:4; [M-H]- | -2.35 | * | -1.47 |  |
| 56 | 13.64 | PE 38:6e; PE 18:2e/20:4; [M-H]- | -2.26 | *** | -2.06 | *** |
| 57 | 13.71 | PE 38:4; PE 18:0-20:4; [M-H]- | -2.13 | * | -2.86 |  |
| 58 | 13.79 | PE 34:3e; PE 16:1e/18:2; [M-H]- | -2.23 | ** | -0.85 |  |
| 59 | 13.83 | PE 38:6e; PE 18:2e/20:4; [M-H]- | -2.33 | * | -2.05 |  |
| 60 | 13.9 | PE 40:7e; PE 18:1e/22:6; [M-H]- | -2.59 | * | -1.36 |  |
| 61 | 13.91 | PE 36:4e; PE 18:2e/18:2; [M-H]- | -2.38 | ** | -1.45 | * |
| 62 | 14.02 | PE 36:5e; PE 16:1e/20:4; [M-H]- | -2.22E+01 |  | -2.47E+01 | * |
| 63 | 14.03 | PE 34:3e; PE 16:1e/18:2; [M-H]- | -1.95 |  | -8.27 |  |
| 64 | 14.07 | PE 38:6e; PE 18:1e/20:5; [M-H]- | -2.35 | * | -2.16 |  |
| 65 | 14.11 | PE 40:7e; PE 18:1e/22:6; [M-H]- | -1.95 |  | 0.67 |  |
| 66 | 14.15 | PE 36:2; PE 18:0-18:2; [M-H]- | 0.05 |  | 4.63 | * |
| 67 | 14.16 | Cer-NS d35:1; Cer-NS d18:1/17:0; [M-H]- | -0.12 |  | 0.32 |  |
| 68 | 14.18 | PE 36:4e; PE 18:2e/18:2; [M-H]- | -2.21 | ** | -1.51 |  |
| 69 | 14.28 | PE 38:4; PE 18:0-20:4; [M-H]- | -1.83 | ** | -1.47 |  |
| 70 | 14.4 | PE 40:6e; PE 18:1e/22:5; [M-H]- | -2.2 | ** | -1.48 | * |
| 71 | 14.43 | PE 38:5e; PE 18:1e/20:4; [M-H]- | -1.48 | * | 1.14 |  |
| 72 | 14.52 | PE 40:7e; PE 18:1e/22:6; [M-H]- | -2.36 | ** | -1.62 |  |
| 73 | 14.57 | PE 36:2; PE 18:0-18:2; [M-H]- | -2.13 | ** | -1.29 |  |
| 74 | 14.58 | PE 40:7e; PE 18:1e/22:6; [M-H]- | -2.39 | ** | -0.76 |  |
| 75 | 14.61 | PE 36:3e; PE 18:1e/18:2; [M-H]- | -1.92 |  | 0.4 |  |
| 76 | 14.72 | Cer-NS d35:1; Cer-NS d18:1/17:0; [M-H]- | -0.41 |  | 0 |  |
| 77 | 14.74 | PE 40:7e; PE 18:1e/22:6; [M-H]- | -2 |  | -2.92E+01 | * |
| 78 | 14.84 | PE 34:0; PE 17:0-17:0; [M-H]- | 0.56 |  | -2.09E+01 |  |
| 79 | 14.86 | PE 40:6e; PE 18:1e/22:5; [M-H]- | -2.35 | ** | -1.22 | ** |
| 80 | 14.91 | PE 38:5e; PE 18:1e/20:4; [M-H]- | -2.2 | ** | -1.3 |  |
| 81 | 14.94 | PE 36:2; PE 18:0-18:2; [M-H]- | -4.05 |  | -4.73 |  |
| 82 | 15.01 | PE 40:6e; PE 18:1e/22:5; [M-H]- | -1.85 | ** | -0.93 | ** |
| 83 | 15.04 | Cer-NS d35:1; Cer-NS d18:1/17:0; [M-H]- | -0.18 |  | -3.18E+01 | * |
| 84 | 15.18 | PE 38:5e; PE 18:1e/20:4; [M-H]- | -1.91 |  | -9.3 | * |
| 85 | 15.19 | PE 38:4e; PE 18:0e/20:4; [M-H]- | -1.46 | ** | -1.36 | ** |
| 86 | 15.2 | PE 36:3e; PE 18:1e/18:2; [M-H]- | -1.87 | ** | -0.59 |  |
| 87 | 15.33 | PE 36:2e; PE 18:0e/18:2; [M-H]- | -2.04 | * | -1.27 |  |
| 88 | 15.46 | PE 36:3e; PE 18:1e/18:2; [M-H]- | -1.92 |  | -6.53 |  |
| 89 | 15.46.1 | PE 34:0; PE 17:0-17:0; [M-H]- | -0.22 |  | 0.19 |  |
| 90 | 15.88 | PE 34:0; PE 17:0-17:0; [M-H]- | 0.63 |  | -7.37 | ** |
| 91 | 16.32 | PE 36:2e; PE 18:1e/18:1; [M-H]- | -1.89 | ** | -0.71 |  |

FC: Fold-change. Color coded according to log_2_(FC) using color bar
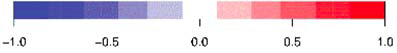
, red represents increased and blue represents decreased concentrations of metabolites. P values corrected by Benjamini & Hochberg method were calculated based on a parametric Student’s t-test or a nonparametric Mann-Whitney test (dependent on the conformity to normal distribution). *P < 0.05, **P < 0.01 and ***P < 0.001

**Table 2 Metabolites and their fold changes from LC-MS positive ion and the associated P-values**

| No. | tR/min | Metabolite name | Effective group (n=33) | | Ineffective group (n=10) | |
| --- | --- | --- | --- | --- | --- | --- |
|  |  |  | FC | P | FC | P |
| 1 | 0.24 | PC 16:0e; PC 14:0e/2:0; [M+H]+ | 0.57 |  | -5.75 |  |
| 2 | 0.33 | PC 18:0e; PC 14:0e/4:0; [M+H]+ | 1.89 |  | -2.73E+01 | * |
| 3 | 0.62 | PC 16:0e; PC 14:0e/2:0; [M+H]+ | 2.11 |  | -7.51 |  |
| 4 | 0.82 | PC 18:0e; PC 14:0e/4:0; [M+H]+ | 1.97 |  | -2.87 |  |
| 5 | 1.1 | ACar 8:1; [M]+ | -2.21 | ** | -0.52 |  |
| 6 | 1.16 | ACar 8:0; [M]+ | -0.6 |  | 0.27 |  |
| 7 | 1.19 | ACar 10:2; [M]+ | -2.25 | ** | -2.45 | ** |
| 8 | 1.2 | ACar 9:0; [M]+ | -0.64 |  | -0.13 |  |
| 9 | 1.25 | ACar 10:1; [M]+ | -0.55 |  | -0.2 |  |
| 10 | 1.28 | ACar 11:1; [M]+ | 0.43 |  | 1.2 | * |
| 11 | 1.34 | ACar 22:6; [M]+ | -1.54 | ** | -0.04 |  |
| 12 | 1.35 | PC 18:0e; PC 14:0e/4:0; [M+H]+ | -1 |  | -2.23E+01 | * |
| 13 | 1.36 | ACar 10:0; [M]+ | 0.01 |  | 0.78 |  |
| 14 | 1.45 | ACar 11:0; [M]+ | -0.46 |  | 0.4 |  |
| 15 | 1.46 | ACar 14:3; [M]+ | -0.95 |  | -0.72 |  |
| 16 | 1.5 | ACar 12:1; [M]+ | -0.42 |  | 0.95 |  |
| 17 | 1.53 | ACar 14:2; [M]+ | 0.25 |  | 0.71 |  |
| 18 | 1.65 | ACar 15:1; [M]+ | 2.68 |  | 2.95 | *** |
| 19 | 1.78 | ACar 12:0; [M]+ | 0.01 |  | 1.23 |  |
| 20 | 1.85 | ACar 14:1; [M]+ | 0.06 |  | 0.6 |  |
| 21 | 1.85.1 | ACar 14:2; [M]+ | -2.17 | ** | -1 |  |
| 22 | 1.88 | ACar 13:0; [M]+ | 0.28 |  | -1.35 |  |
| 23 | 1.88.1 | PC 20:5e; PC 18:5e/2:0; [M+H]+ | 0.49 |  | 0.79 |  |
| 24 | 2.09 | ACar 14:1; [M]+ | -0.02 |  | 0.72 |  |
| 25 | 2.32 | LPC 20:5; [M+H]+ | 0.6 |  | 0.36 |  |
| 26 | 2.37 | LPC 18:3; [M+H]+ | 0.17 |  | 0.83 |  |
| 27 | 2.38 | ACar 17:1; [M]+ | 2.92 | ** | 3.95 | *** |
| 28 | 2.4 | LPC 14:0; [M+H]+ | -0.46 |  | 0.27 |  |
| 29 | 2.4.1 | LPC 18:3; [M+H]+ | 0.11 |  | 1.05 |  |
| 30 | 2.41 | PC 16:1e; PC 14:1e/2:0; [M+H]+ | 0.81 |  | 2.97 |  |
| 31 | 2.64 | ACar 18:3; [M]+ | -0.5 |  | -0.23 |  |
| 32 | 2.75 | LPC 15:0; [M+H]+ | 0.53 |  | 1.66 |  |
| 33 | 2.75.1 | PC 16:1e; PC 14:1e/2:0; [M+H]+ | -0.41 |  | 0.98 |  |
| 34 | 2.75.2 | PC 20:5e; PC 18:5e/2:0; [M+H]+ | 0.87 |  | 1.14 | * |
| 35 | 2.79 | LPC 20:4; [M+H]+ | 0.83 |  | 1.22 | ** |
| 36 | 2.83 | LPC 18:2; [M+H]+ | 1.04 |  | 2.54 | *** |
| 37 | 2.98 | PC 20:4e; PC 18:4e/2:0; [M+H]+ | 0.95 |  | 1.9 | *** |
| 38 | 3.01 | ACar 16:1; [M]+ | -0.04 |  | 0.79 |  |
| 39 | 3.03 | LPC 15:0; [M+H]+ | -0.29 |  | 0.94 |  |
| 40 | 3.06 | ACar 18:2; [M]+ | 2.58 |  | 9.42 | * |
| 41 | 3.06.1 | LPE 17:1; [M+H]+ | 0.08 |  | 0.44 |  |
| 42 | 3.08 | LPE 18:2; [M+H]+ | -1.22 | ** | -0.21 |  |
| 43 | 3.08.1 | PC 17:1e; PC 14:1e/3:0; [M+H]+ | 0.05 |  | 3.54 |  |
| 44 | 3.11 | LPC 16:0; [M+H]+ | 1.58 |  | 6.29 |  |
| 45 | 3.12 | LPC 15:0; [M+H]+ | -0.51 |  | 0.42 |  |
| 46 | 3.12.1 | PC 18:2e; PC 16:2e/2:0; [M+H]+ | 0.79 |  | 3.08 | *** |
| 47 | 3.26 | PC 20:4e; PC 18:4e/2:0; [M+H]+ | 1.23 |  | 1.13 |  |
| 48 | 3.3 | LPE 17:1; [M+H]+ | -0.09 |  | 0.73 |  |
| 49 | 3.37 | LPE 18:2; [M+H]+ | -1.34 |  | -6.14 | * |
| 50 | 3.39 | ACar 20:4; [M]+ | -0.33 | * | 1.23 |  |
| 51 | 3.45 | PC 17:1e; PC 14:1e/3:0; [M+H]+ | -0.35 |  | 0.29 |  |
| 52 | 3.47 | PC 16:0e; PC 14:0e/2:0; [M+H]+ | -0.14 |  | 0.54 |  |
| 53 | 3.5 | ACar 18:2; [M]+ | -0.48 |  | 0.96 |  |
| 54 | 3.53 | LPC 18:2; [M+H]+ | -0.84 |  | -2.68 |  |
| 55 | 3.63 | LPE 17:1; [M+H]+ | -0.09 |  | -6.66 | * |
| 56 | 3.85 | ACar 18:2; [M]+ | -0.12 |  | -6.1 | ** |
| 57 | 3.85.1 | LPC 16:0; [M+H]+ | -0.31 | * | 0.69 |  |
| 58 | 3.86 | ACar 16:0; [M]+ | 0.23 |  | 30.69 |  |
| 59 | 3.87 | PC 17:1e; PC 14:1e/3:0; [M+H]+ | 0.39 |  | -1.93 |  |
| 60 | 3.89 | PC 18:1e; PC 16:1e/2:0; [M+H]+ | 0.26 |  | 3.09 | *** |
| 61 | 4.15 | PC 16:0e; PC 14:0e/2:0; [M+H]+ | 0.39 |  | -5.02 |  |
| 62 | 4.28 | ACar 18:1; [M]+ | 0.56 |  | 0.37 |  |
| 63 | 4.33 | PC 18:1e; PC 16:1e/2:0; [M+H]+ | 0.26 |  | 1.46 | * |
| 64 | 4.34 | ACar 16:0; [M]+ | -0.34 |  | 0.57 |  |
| 65 | 4.34.1 | LPE 18:1; [M+H]+ | -1.47 |  | -0.57 |  |
| 66 | 4.43 | PC 17:0e; PC 14:0e/3:0; [M+H]+ | -0.19 |  | 0.45 |  |
| 67 | 4.61 | ACar 16:0; [M]+ | 0.15 |  | -6.55 |  |
| 68 | 4.64 | LPE 18:1; [M+H]+ | -0.72 |  | -2.81 |  |
| 69 | 4.68 | PC 18:1e; PC 16:1e/2:0; [M+H]+ | 0.48 |  | -3.61 |  |
| 70 | 4.8 | ACar 18:1; [M]+ | -0.28 |  | 0.68 |  |
| 71 | 4.9 | LPE 17:0; [M+H]+ | -0.04 |  | 1.14 |  |
| 72 | 4.92 | LPC 17:0; [M+H]+ | -0.36 |  | 0.88 |  |
| 73 | 5.08 | PC 18:0e; PC 16:0e/2:0; [M+H]+ | 0.32 |  | 7.07 | * |
| 74 | 5.13 | ACar 18:1; [M]+ | -0.09 |  | -5.6 | * |
| 75 | 5.28 | PC 16:0e; PC 14:0e/2:0; [M+H]+ | -0.02 |  | -0.53 |  |
| 76 | 5.28.1 | PC 17:0e; PC 14:0e/3:0; [M+H]+ | -0.07 |  | -4.75 | ** |
| 77 | 5.57 | LPC 18:0; [M+H]+ | -0.27 |  | 0.35 |  |
| 78 | 5.58 | LPE 18:0; [M+H]+ | -1.43 | * | 0.94 |  |
| 79 | 5.76 | LPC 18:2; [M+H]+ | 0.6 |  | 1.1 |  |
| 80 | 6.09 | PC 18:0e; PC 16:0e/2:0; [M+H]+ | -0.15 |  | 0.8 |  |
| 81 | 6.21 | LPC 17:0; [M+H]+ | -0.11 |  | -0.18 |  |
| 82 | 6.21.1 | PC 18:2e; PC 16:2e/2:0; [M+H]+ | 0.62 |  | 1.57 | * |
| 83 | 6.48 | PC 18:0e; PC 16:0e/2:0; [M+H]+ | -0.23 |  | -4.05 | * |
| 84 | 6.56 | ACar 18:0; [M]+ | -0.51 | * | 0.22 |  |
| 85 | 6.7 | PC 16:0e; PC 14:0e/2:0; [M+H]+ | -0.28 |  | 0.01 |  |
| 86 | 6.9 | ACar 18:0; [M]+ | -0.3 |  | -6.12 |  |
| 87 | 7.22 | PC 16:0e; PC 14:0e/2:0; [M+H]+ | 0.28 |  | 0.39 |  |
| 88 | 7.36 | PC 19:0e; PC 14:0e/5:0; [M+H]+ | -0.1 |  | 0.23 |  |
| 89 | 7.69 | LPC 18:0; [M+H]+ | 0.26 |  | -0.1 |  |
| 90 | 7.69.1 | PC 19:0e; PC 14:0e/5:0; [M+H]+ | 0.04 |  | -2.99 |  |
| 91 | 7.73 | PC 16:0e; PC 14:0e/2:0; [M+H]+ | 0.07 |  | 0.11 |  |
| 92 | 8.48 | PC 18:2e; PC 16:2e/2:0; [M+H]+ | 1.17 | * | 0.33 |  |
| 93 | 8.68 | PC 16:0e; PC 14:0e/2:0; [M+H]+ | -0.36 |  | -1.69 |  |
| 94 | 8.71 | LPC 18:0; [M+H]+ | -0.03 |  | -0.83 |  |
| 95 | 8.72 | LPC 18:1; [M+H]+ | -0.08 |  | -1.1 |  |
| 96 | 9.13 | PC 16:0e; PC 14:0e/2:0; [M+H]+ | -0.59 |  | -2.55 | * |
| 97 | 9.46 | LPC 17:0; [M+H]+ | -0.53 |  | -1.83 |  |
| 98 | 9.56 | PC 18:0e; PC 16:0e/2:0; [M+H]+ | -0.54 |  | -0.72 |  |
| 99 | 9.57 | PC 16:0e; PC 14:0e/2:0; [M+H]+ | -0.41 |  | -0.45 |  |
| 100 | 9.98 | PC 16:0e; PC 14:0e/2:0; [M+H]+ | -0.56 |  | -0.24 |  |
| 101 | 10.32 | LPC 18:0; [M+H]+ | -0.4 |  | -0.07 |  |
| 102 | 10.37 | PC 16:0e; PC 14:0e/2:0; [M+H]+ | -0.24 |  | -0.95 |  |
| 103 | 10.78 | LPC 18:0; [M+H]+ | -0.34 |  | -0.47 |  |
| 104 | 10.83 | ACar 24:0; [M]+ | -0.31 |  | 0.11 |  |
| 105 | 10.86 | PC 16:0e; PC 14:0e/2:0; [M+H]+ | -0.47 |  | -0.4 |  |
| 106 | 11.03 | DAG 24:0; DAG 12:0-12:0; [M+NH4]+ | -0.49 |  | 0.17 |  |
| 107 | 11.23 | PC 18:2e; PC 16:2e/2:0; [M+H]+ | 0.43 |  | 0.7 |  |
| 108 | 11.36 | PC 18:0e; PC 16:0e/2:0; [M+H]+ | 0.05 |  | -0.59 |  |
| 109 | 11.42 | PC 16:0e; PC 14:0e/2:0; [M+H]+ | 0.31 |  | -0.73 |  |
| 110 | 11.6 | LPC 17:0; [M+H]+ | 0.57 |  | -0.26 |  |
| 111 | 11.65 | PC 20:4e; PC 18:4e/2:0; [M+H]+ | 24.58 | * | -2.32E+01 |  |
| 112 | 12.02 | PC 16:0e; PC 14:0e/2:0; [M+H]+ | 0.28 |  | -1.48 |  |
| 113 | 12.09 | PC 18:0e; PC 16:0e/2:0; [M+H]+ | 0.17 |  | 0.79 |  |
| 114 | 12.14 | PC 18:2e; PC 16:2e/2:0; [M+H]+ | 1.46 |  | 1.41 |  |
| 115 | 12.62 | PC 18:0e; PC 16:0e/2:0; [M+H]+ | 0.2 |  | 0.18 |  |
| 116 | 12.63 | LPC 16:0; [M+H]+ | -0.34 |  | -0.4 |  |
| 117 | 12.69 | PC 17:0e; PC 14:0e/3:0; [M+H]+ | -1.55 |  | -1.24 |  |
| 118 | 13.01 | PC 16:0e; PC 14:0e/2:0; [M+H]+ | -0.7 |  | -1 |  |
| 119 | 13.59 | LPC 18:0; [M+H]+ | -0.29 |  | -0.09 |  |
| 120 | 14.12 | LPC 18:0; [M+H]+ | -0.17 |  | -0.99 |  |
| 121 | 14.22 | LPC 16:0; [M+H]+ | -0.8 |  | 0.21 |  |
| 122 | 14.73 | PC 16:0e; PC 14:0e/2:0; [M+H]+ | -1.48 |  | 0.18 |  |
| 123 | 18.88 | PC 16:0e; PC 14:0e/2:0; [M+H]+ | -0.06 |  | -0.45 |  |
| 124 | 21.76 | PC 16:0e; PC 14:0e/2:0; [M+H]+ | 0.52 |  | -0.67 |  |
| 125 | 28.76 | LPC 18:0; [M+H]+ | 0.44 |  | -2 |  |
| 126 | 29.33 | LPC 18:0; [M+H]+ | -0.03 |  | 0.23 |  |
| 127 | 29.74 | PC 18:0e; PC 16:0e/2:0; [M+H]+ | -0.21 |  | 0.31 |  |
| 128 | 29.76 | LPC 16:0; [M+H]+ | -0.8 |  | -0.65 |  |

FC: Fold-change. Color coded according to log_2_(FC) using color bar
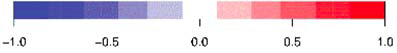
, red represents increased and blue represents decreased concentrations of metabolites. P values corrected by Benjamini & Hochberg method were calculated based on a parametric Student’s t-test or a nonparametric Mann-Whitney test (dependent on the conformity to normal distribution). *P < 0.05, **P < 0.01 and ***P < 0.001

**Table 3 Metabolites and their fold changes from GC-MS and the associated P-values**

| No. | Metabolite name | Effective group (n=33) | | Ineffective group (n=10) | |
| --- | --- | --- | --- | --- | --- |
|  |  | FC | P | FC | P |
| 1 | Para-cymene | -1.76E+01 | *** | 0 | * |
| 2 | Phenobarbital | -1.64E+01 | *** | 0 | * |
| 3 | Cyclobarbital | -1.50E+01 | *** | -0.01 | * |
| 4 | gamma-Linolenic acid | -1.87E+01 | ** | -1.93E+01 |  |
| 5 | Hypotaurine | -1.86E+01 | *** | 0 |  |
| 6 | p-Aminohippuric acid | 19.77 |  | 16.37 |  |
| 7 | Fructose-6-phosphate | -0.01 | *** | 0 | * |
| 8 | 3-Amino-2,3-dihydrobenzoic acid | -1.52E+01 | *** | -1.51E+01 | *** |
| 9 | L-Homocitrulline | -1.66E+01 | *** | -1.45E+01 | *** |
| 10 | DL-Norvaline | 1.07 | ** | 15.81 | ** |
| 11 | Urea | 0 | *** | 0 | * |
| 12 | Hydroxylamine | -1.86E+01 |  | 21.43 |  |
| 13 | Cystamine | -1.74E+01 | *** | -1.72E+01 | *** |
| 14 | DL-beta-Hydroxybutyric acid | -0.01 | *** | 0 | * |
| 15 | L-Valine | 4.98 | * | -1.65E+01 |  |
| 16 | N-Methyl-DL-Alanine | -1.15 |  | -1.19 |  |
| 17 | 3-Methyladenine | -4.97 | * | -3.14 |  |
| 18 | Cysteinylglycine | -12.51 | *** | 0 | * |
| 19 | N-Carbamoyl-L-Aspartate | -0.01 | *** | 0 | * |
| 20 | N-Piperoyldiethlamine | -1.77E+01 | *** | -1.67E+01 | * |
| 21 | Ferulic acid, cis- | -1.68E+01 | *** | 0 | * |
| 22 | 2-Hydroxypyridine | -12.5 | *** | -12.55 | *** |
| 23 | 3-Hydroxypyruvate | 0 | *** | -1.37E+01 | *** |
| 24 | L-(+)-Lactic acid | 0 | *** | 0 | * |
| 25 | D-Ribulose | -11.59 | *** | 0 |  |
| 26 | Glycolic acid | -11.42 | *** | -11.34 | ** |
| 27 | L-Alanine | 1.33 |  | 3.91 |  |
| 28 | (R)-(-)-Phenylephrine | -1.95 |  | -1.89E+01 |  |
| 29 | 2-Hydroxyisobutyric acid | -1.48E+01 | *** | -1.50E+01 | *** |
| 30 | 3,4-Dihydroxy-L-phenylalanine | -1.41E+01 | *** | -1.39E+01 | *** |
| 31 | 5-Fluorouracil | -12.57 | *** | -12.37 | *** |
| 32 | L-3,4-Dihydroxymandelic acid | -11.05 | *** | 0 | * |
| 33 | L-Prolinamide | -1.42E+01 | *** | -12.35 | ** |
| 34 | Ethylene Glycol Diacetate | -0.01 | *** | -0.01 |  |
| 35 | DL-beta-Hydroxybutyric acid (2TMS) | -1.39E+01 | *** | -1.44E+01 | *** |
| 36 | Pyridoxamine | -1.44E+01 | *** | -1.43E+01 | *** |
| 37 | Alanylalanine | -12.81 | *** | -1.33E+01 | *** |
| 38 | L-Cysteic acid | -1.40E+01 | *** | -1.43E+01 | *** |
| 39 | Putrescine | -0.01 | *** | 0 |  |
| 40 | Succinic semialdehyde | -11.76 | *** | 0.01 |  |
| 41 | Phosphoenolpyruvic acid | -13.25 | *** | -13.01 | ** |
| 42 | L-Norvaline | 2.21 | *** | 15.23 | ** |
| 43 | Indole-3-acetic acid | -13.27 | *** | -1.41E+01 | *** |
| 44 | L-Leucine | -12 | *** | -11.32 | ** |
| 45 | 3-Phenylpropionic acid | -1.71E+01 | *** | -1.68E+01 | *** |
| 46 | Homocystine | -10.98 | *** | -10.42 | ** |
| 47 | Glyceric acid | -1.21 |  | -2.49 |  |
| 48 | L-Asparagine | -1.51E+01 | *** | -1.52E+01 | *** |
| 49 | Pyrophosphate | 0.33 |  | 0.17 |  |
| 50 | D-(+)-Fucose | -1.43E+01 | *** | -1.43E+01 | *** |
| 51 | L-Proline | 14.98 | *** | 15.56 | ** |
| 52 | Dopamine | -1.73E+01 | *** | -1.54E+01 | ** |
| 53 | Succinic acid | -11.88 | *** | -12.4 | *** |
| 54 | Phthalic acid | -1.69 |  | -1.76 | * |
| 55 | Uracil | 0 | *** | 0 | * |
| 56 | L-(+)-Tartarate | -1.43E+01 | *** | -1.48E+01 | *** |
| 57 | Taurine | 0 | *** | -10.91 | ** |
| 58 | Dihydroxyacetone | -1.56E+01 | *** | -1.60E+01 | *** |
| 59 | Fumaric acid | -11.81 | *** | -12.47 | *** |
| 60 | Nonanoic acid | -1.46E+01 | *** | -1.45E+01 | *** |
| 61 | 4-Methyl Benzoic acid | -1.82E+01 | *** | -1.85E+01 |  |
| 62 | L-Threo-b-HydroxyAspartic acid | -0.41 |  | 0.43 |  |
| 63 | DethioBiotin | -1.33E+01 | *** | -13.2 | *** |
| 64 | DL-Threo-b-HydroxyAspartic acid | -0.52 | ** | 0.39 |  |
| 65 | 5-Aminovaleric acid | -1.45E+01 | *** | -1.39E+01 |  |
| 66 | Myo-Inositol | -12.75 | *** | -12.93 | ** |
| 67 | 2'-Deoxyadenosine | -13.28 | *** | -1.38E+01 | ** |
| 68 | Methyl Dopa | 0.41 |  | 0.72 | * |
| 69 | L-Pyroglutamic acid | -1.82E+01 | ** | -1.83E+01 |  |
| 70 | Adenine | -1.57 |  | -1.67 |  |
| 71 | Creatinine | 0 | ** | 0 | * |
| 72 | Gluconic acid | -12.54 | *** | 0 | * |
| 73 | Uridine | -0.01 | *** | -10.31 | ** |
| 74 | 2-Thiouracil | -12.45 | *** | -11.77 | * |
| 75 | L-Glutamic acid | 0 |  | 1.81 | * |
| 76 | L-Tyrosine | -0.36 | ** | 0.87 | * |
| 77 | alpha-Lactose | -1.48E+01 | *** | -1.50E+01 | *** |
| 78 | Galactitol | -11.9 | *** | -12.35 | *** |
| 79 | Xylitol | 0 |  | -11.71 |  |
| 80 | Xanthine | -13.17 | *** | -1.34E+01 | *** |
| 81 | D-Arabitol | -12.98 | *** | -13.06 | *** |
| 82 | D-Ribulose-5-phosphate | -3 |  | -4.11 |  |
| 83 | 4-Hydroxyphenethyl alcohol | -12.55 | *** | -1.34E+01 | *** |
| 84 | Benzen-1,3-Dicarboxylic acid | -11.37 | *** | -11.44 | *** |
| 85 | n-Caprylic acid | -12.23 | *** | -11.95 | ** |
| 86 | Citric acid | -2.73 |  | -2.19 |  |
| 87 | 3,4-Dihydroxybenzoic acid | -1.59E+01 | ** | -1.61E+01 |  |
| 88 | 1-Kestose | -1.60E+01 | *** | -4.61 |  |
| 89 | 1,5-Anhydro-D-glucitol | -3.26 |  | -3.13 |  |
| 90 | Icosanoic acid | -1.50E+01 | *** | -1.46E+01 | * |
| 91 | L-Lysine | 0 |  | 12.35 |  |
| 92 | beta-D-(+)-Glucose | -1.51E+01 | ** | -1.58E+01 | ** |
| 93 | D-(-)-Sorbitol | -1.47E+01 | *** | -1.45E+01 | ** |
| 94 | D-(+)-Allose | 20.27 |  | 21.38 |  |
| 95 | D-(+)-Galactose | -0.1 |  | 19.13 | ** |
| 96 | D-Glucarate | -11.38 | *** | -13.15 | ** |
| 97 | L-Glucono-1,4-lactone | -12.65 | *** | -12.49 | ** |
| 98 | 2,6-Diaminopurine | -1.50E+01 | *** | -1.52E+01 | *** |
| 99 | Glycerol 1-phosphate | -11.56 | *** | -11.79 | ** |
| 100 | Maltose | -12.66 | *** | -1.45E+01 | *** |
| 101 | L-Tryptophane | -11.94 | *** | -12.26 | ** |
| 102 | Palmitoleate | 0 | *** | -12.84 | *** |
| 103 | Xanthosine | -10.15 | *** | -0.01 | * |
| 104 | Heptadecanoate | -1.37E+01 | *** | -1.34E+01 | ** |
| 105 | 2-Dehydro-D-gluconate | -12.79 | *** | -13.15 | ** |
| 106 | D-Arabinose-5-phosphate | 18.97 | * | 18.85 | *** |
| 107 | Oleic acid | -0.97 |  | -0.39 |  |
| 108 | Elaidic acid | -1.36E+01 | *** | -1.42E+01 | ** |
| 109 | D-Fructose-6-Phosphate | -11.75 | *** | -13.01 | *** |
| 110 | 5-Hydroxymethyl Uracil | -1.50E+01 | *** | -1.53E+01 | *** |
| 111 | L-Valine | -10.89 | *** | -11.35 | ** |
| 112 | D-Sorbitol-6-phosphate | -1.44E+01 | *** | -1.47E+01 | *** |
| 113 | Homogentisate | 15.83 | * | 2.46 | ** |
| 114 | L-Gulcono-1,4-lactone | -0.01 | *** | 0 | * |
| 115 | L-Saccharopine | 17.11 | * | 16.66 | *** |
| 116 | 4-Hydroxypyridine | -13.18 | *** | -1.37E+01 | *** |
| 117 | Pyrogallol | 0.12 |  | 19.57 |  |
| 118 | D-(+)-Trehalose | -1.46E+01 | *** | -1.49E+01 | *** |
| 119 | Lignoceric acid | -10.84 | *** | -10.82 | ** |
| 120 | Maltotriose | -11.82 | *** | -12.15 | *** |
| 121 | 16-Hydroxyhexadecanoic acid | -12.76 | *** | -13.02 | *** |
| 122 | alpha-Lactose (8TMS) | -13.28 | *** | -12.8 | *** |
| 123 | D-(+)-Maltose | -1.47E+01 | *** | -1.46E+01 | *** |
| 124 | Adenosine 3':5'-cyclicmonophosphate | -13.17 | *** | -1.34E+01 | *** |
| 125 | Benzen<U+2212>1,2<U+2212>Dicarboxylic acid | -11.15 | *** | -11.36 | *** |
| 126 | Octacosanoic acid | -10.62 | *** | -10.97 | *** |
| 127 | (+-)-alpha-Tocopherol | -1.40E+01 | * | -1.51E+01 | *** |
| 128 | 2-MethylHippurate | -11.08 | *** | -9.2 | * |
| 129 | Inositol-1-phosphate, myo- | 0 |  | -0.52 | * |
| 130 | 2'-Deoxyadenosine (3TMS) | -3.67 |  | -1.82E+01 |  |
| 131 | Glycerol | 0 | *** | 0 | * |

FC: Fold-change. Color coded according to log_2_(FC) using color bar
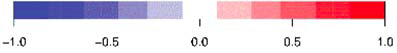
, red represents increased and blue represents decreased concentrations of metabolites. P values corrected by Benjamini & Hochberg method were calculated based on a parametric Student’s t-test or a nonparametric Mann-Whitney test (dependent on the conformity to normal distribution). *P < 0.05, **P < 0.01 and ***P < 0.001
